# Supplementary material for: Transcriptomic Dysregulation of Inflammation-Related Genes in Leukocytes of Patients with Gestational Diabetes Mellitus (GDM) during and after Pregnancy: Identifying Potential Biomarkers Relevant to Glycemic Abnormality
Source: Int J Mol Sci. 2022 Nov 24;23(23):14677. doi: 10.3390/ijms232314677 (PMC9737950; doi:10.3390/ijms232314677)
Supplement: Supplementary file 1 [file ijms-23-14677-s001.zip › ijms-1979409-supplementary.pdf]

Table S1. PCR primer list

GenBank	Gene	Description		Sequence 5′->3′	Product length [bp]
NM_000600.5	*IL6*	Interleukin 6	OF:	CATCCTCGACGGCATCTCAG	163
NM_001371096.1		(interferon, beta 2)	OR:	CACCAGGCAAGTCTCCTCATT	
			IF:	CCTGAGAAAGGAGACATGTAACAAG	79
			IR:	AAGGTTCAGGTTGTTTTCTGCC	
NM_000584.4	*CXCL8*	Interleukin 8 (IL8, C-	OF:	TTTTGCCAAGGAGTGCTAAAGA	194
NM_001354840.3		X-C motif chemokine	OR:	AACCCTCTGCACCCAGTTTTC	
		ligand 8, CXCL8)	IF:	ACTGAGAGTGATTGAGAGTGGAC	112
			IR:	AACCCTCTGCACCCAGTTTTC	
NM_000572.3	*IL10*	Interleukin 10	OF:	CGAGATGCCTTCAGCAGAGT	257
		(cytokine synthesis	OR:	AGAAATCGATGACAGCGCC	
		inhibitory factor,	IF:	CTTTAAGGGTTACCTGGGTTGC	103
		IL10A,TGIF,GVHDS,)	IR:	CGCCTTGATGTCTGGGTCTT	
NM_001354991.2	*IL13*	Interleukin 13	OF:	CTGGAATCCCTGATCAACGTGT	322
NM_001354992.2			OR:	TTTACCCCTCCCTAACCCTCC	
NM_001354993.2			IF:	AAGGTCTCAGCTGGGCAGTTTT	115
NM_002188.3			IR:	ACTGTCCCTCGCGAAAAAGTT	
NM_001243211.2	*IL18*	Interleukin 18	OF:	AAGATGGCTGCTGAACCAGT	245
NM_001386420.1		(interferon-gamma-	OR:	GTCCGGGGTGCATTATCTCT	
NM_001562.4		inducing factor)	IF:	GCTGAAGATGATGAAAACCTGGA	115
			IR:	GAGGCCGATTTCCTTGGTCA	
NM_001145138.2	*RELA*	Polypeptide gene	OF:	CTCCTGTGCGTGTCTCCAT	214
NM_001243984.2		enhancer in B-cells 3,	OR:	GAAGGCACAGCAATGCGTC	
NM_001243985.2		p65	IF:	GCCAGATACAGACGATCGTCA	138
NM_021975.4			IR:	GAAGGCACAGCAATGCGTC	
NM_000594.4	*TNFA*	Tumor necrosis factor	OF:	GAGGCGCTCCCCAAGAAGAC	416
		(TNF superfamily,	OR:	GTGAGGAGCACATGGGTGGA	
		member 2)	IF:	GCCTCTTCTCCTTCCTGATCG	143
			IR:	AGAAGATGATCTGACTGCCTGG	
NM_001101.5	*ACTB*	Actin beta	OF:	GCCGAGACCGCGTCC	186
			OR:	CACGATGGAGGGGAAGACG	
			IF:	GCACAGAGCCTCGCCTT	93
			IR:	GTTGTCGACGACGAGCG	

Abbreviations: bp, base pairs; IF, inner forward; IR, inner reverse; OF, outer forward; OR, outer reverse

Table S2. Logistic regression models for predicting postpartum AGT in GDM patients (postpartum NGT, n=18; postpartum AGT, n=10)

		**Global significancy *p-value***			**Goodness of fit**							**Validation**	
**Model no**	**Factors**	**LR test**	**Wald's test**	**Hosmer Lemeshow's test *p-value***	**AIC**	**AICC**	**BIC**	**Cox-Snell's R2**	**Nagelkerk's R2**	**AUC**	**AUC Error**	**AUC**	**AUC Error**
1	*IL8*	**0.0195**	**0.0370**	0.4628	35.04	35.52	37.71	0.18	0.24	0.75	0.11	0.65	0.12
2	*IL13*	**0.0356**	0.0542	0.1696	36.08	36.56	38.75	0.15	0.20	0.73	0.10	0.52	0.12
3	*TNFA*	**0.0097**	**0.0273**	0.2820	33.80	34.28	36.47	0.21	0.29	0.76	0.10	0.66	0.13
4	*FPG*	0.2161	0.2287	0.6225	38.97	39.45	41.63	0.05	0.07	0.71	0.10	0.54	0.13
5	*IL8*+*IL13*+*TNFA*+FPG	**0.0030**	**0.1004**	0.6381	30.52	33.24	37.18	0.43	**0.60**	**0.91**	**0.06**	**0.79**	**0.10**
6	*IL8*+*IL13*+*TNFA*	**0.0013**	0.0536	0.6665	28.75	30.49	34.08	0.43	0.59	0.91	0.06	0.78	0.10
7	*IL8xTNFA*+*IL13*	**0.0002**	**0.0336**	0.5174	**25.19**	**26.19**	**29.18**	**0.46**	**0.63**	**0.89**	**0.06**	0.68	0.13
8	*IL8*+*IL13*	**0.0030**	**0.0328**	0.4821	30.87	31.87	34.87	0.34	0.47	**0.87**	**0.07**	**0.77**	**0.09**
9	[*IL8xIL13xTNFA*xFPG]	**0.0001**	**0.0178**	0.3531	**25.45**	**25.93**	**28.11**	**0.42**	**0.57**	0.85	0.08	0.76	0.12
10	[*IL8xIL13xTNFA*]	**0.0002**	**0.0140**	0.3925	**26.56**	**27.04**	**29.22**	**0.39**	0.54	0.85	0.08	**0.80**	0.11

The significant results of statistical tests and best scores of each goodness of fit measurement shown in bold.

Abbreviations: AIC, Akaike Information Criterion; AICC, Akaike Information Corrected Criterion; AUC, area under the curve BIC, Bayesian Information Criterion; LR test, Likelihood-Ratio test
